# Simian Varicella Virus DNA in Saliva and Buccal Cells After Experimental Acute Infection in Rhesus Macaques

**DOI:** 10.3389/fmicb.2019.01009

**Published:** 2019-05-09

**Authors:** Vicki Traina-Dorge, Satish Mehta, Bridgette Rooney, Brian Crucian, Lara Doyle-Meyers, Arpita Das, Colin Coleman, Maria Nagel, Ravi Mahalingam

**Affiliations:** ^1^ Division of Microbiology, Tulane National Primate Research Center, Tulane University, Covington, LA, United States; ^2^ Jes Tech, Houston, TX, United States; ^3^ KBR wyle Laboratories, Houston, TX, United States; ^4^ GeoControl Systems, Inc., Houston, TX, United States; ^5^ Johnson Space Center, NASA, Houston, TX, United States; ^6^ Division of Veterinary Medicine, Tulane National Primate Research Center, Tulane University, Covington, LA, United States; ^7^ Department of Neurology, University of Colorado Anschutz Medical Campus, Aurora, CO, United States; ^8^ Department of Ophthalmology, University of Colorado Anschutz Medical Campus, Aurora, CO, United States

**Keywords:** simian varicella virus, rhesus macaque, acute infection, saliva, buccal cells

## Abstract

Simian varicella virus (SVV) infection of non-human primates is the counterpart of varicella zoster virus (VZV) infection in humans. To develop non-invasive methods of assessing SVV infection, we tested for the presence of SVV DNA in saliva, as has been documented in human VZV infection, and in buccal cells to determine whether epithelial cells might provide a more reliable source of material for analysis. Five rhesus macaques intratracheally inoculated with SVV all developed varicella with viremia and macular-papular skin rash in 1–2 weeks, which resolved followed by establishment of latency. DNA extracted from longitudinal blood peripheral blood mononuclear cells (PBMCs), saliva and buccal samples collected during acute infection and establishment of latency were analyzed by real-time qPCR. After intratracheal inoculation, viremia was observed, with peak levels of 10^1^–10^2^ copies of SVV DNA in 100 ng of PBMC DNA at 4 and 7 days post inoculation (dpi), which then decreased at 9–56 dpi. In saliva and buccal cells at 7 dpi, 10^1^–10^4^ copies and 10^1^–10^5^ copies of SVV DNA in 100 ng of cellular DNA, respectively, were detected in all the five monkeys. At 9 dpi, saliva samples from only two of the five monkeys contained SVV DNA at 10^2^–10^3^ copies/100 ng of saliva DNA, while buccal cells from all five monkeys showed 10^0^–10^3^ copies of SVV DNA/100 ng of buccal cell DNA. Similar to viremia, SVV DNA in saliva and buccal cells at 11–56 dpi was lower, suggesting clearance of viral shedding. SVV DNA levels were generally higher in buccal cells than in saliva. Our findings indicate that SVV shedding into the oral cavity parallels acute SVV infection and underscore the relevance of both saliva and buccal cell samples to monitor acute varicella virus infection.

## Introduction

Simian varicella virus (SVV) infection of non-human primates (NHP) is the simian counterpart of varicella zoster virus (VZV) infection in humans. Similar to primary VZV infection in humans, NHPs experimentally infected with SVV, develop acute varicella with malaise, fever, viremia, and generalized vesicular rash (Messaoudi et al., 2009; Ouwendijk et al., 2013). Following acute SVV infection, the virus establishes latency and later reactivates to produce zoster (Mahalingam et al., 2007, 2010; James et al., 2014; Traina-Dorge et al., 2014). As such, the SVV infection in NHP has been an extremely valuable animal model to further study the pathobiology and neurotropism of VZV showing virus entering ganglia before the appearance of primary rash; viral transport is *via* both hematogenous and non-hematogenous routes, critical gene expression, and cell signaling changes associated with disease; tropism of ganglionic, epithelial, and T lymphocytes (Mahalingam et al., 2001; Ouwendijk et al., 2013; Traina-Dorge et al., 2015, 2019; Arnold et al., 2016).

Our goal here was the development of a non-invasive method to analyze SVV infection and viremia after acute infection in non-human primates. We attempted to detect SVV DNA in saliva, as has been performed in VZV infection in adult humans (Mehta et al., 2012), in patients with zoster (Mehta et al., 2008, 2013, 2017; Depledge et al., 2011; Nagel et al., 2011; Gershon et al., 2015), and in patients with zoster sine herpete (Furuta et al., 2001; Gershon et al., 2015). We also analyzed buccal cells for SVV DNA to determine whether buccal epithelial cells from anesthetized animals could provide a more reliable source of material.

## Materials and Methods

### Animals

Five male rhesus macaques (KB95, KG58, KI87, KI92, and KT89), *Macaca mulatta*, (4–5 kg) were prescreened for SVV-seronegativity using plaque reduction antibody neutralization assays. All animals were housed at the Tulane National Primate Research Center in Covington, LA. On day 0, baseline physical examinations were performed, and blood, saliva, and buccal samples were obtained, while the ventrum and appendages of the animals were shaved. All the five animals were then intratracheally inoculated with 5 × 10^5^ pfu (plaque forming units) wild type SVV (wtSVV) in 1 ml using a sterile #10 French feeding tube. SVV was grown in Rhesus fibroblast monolayers and cell-associated virus harvested for inoculation. After SVV inoculation, all monkeys were monitored by physical examination and collection of blood, saliva, and buccal samples, 2–3 times for the first 2 weeks, weekly for 8 weeks, and monthly thereafter. Animals were evaluated for respiratory, cardiac, lymphatic, or dermal involvement, and any adverse findings were documented. Typical varicella lesions (macules, papules, and vesicles) were scored as follows: +/− 1–2; 1+: 3–10; 2+: 11–50; 3+: 51–100; 4+: >101 lesions. All examinations and procedures were performed following anesthesia of the animals with 10 mg/kg intramuscular ketamine hydrochloride or 6–8 mg/kg intramuscular Tiletamine/zolazepam (Telazol) in accordance with the recommendations of the US Department of Agriculture Animal Welfare Act regulations, the *Guide for the Care and Use of Laboratory Animals* and regulations of the Association for Assessment and Accreditation of Laboratory Animal Care (AAALAC). All experiments were reviewed and approved by the Tulane National Primate Research Center (TNPRC) Institutional Animal Care and Use Committee (IACUC), prior to the start of the study.

### Saliva and Buccal Swab Collection

**Saliva—**Synthetic polymer saliva swabs, SalivaBio (Salimetrics, State College, PA), were placed for 3 min each in the left and right cheek pouches of each anesthetized monkey to saturate with saliva. The swabs were removed, transferred to the compartmentalized collection tube, and placed on ice. Swabs and tubes were centrifuged at 5,000 × g at 4°C for 20 min and saliva was recovered in the bottom chamber. The volume of saliva (50–1,200 μl) was recorded and transferred to 1.5 ml microcentrifuge tubes. Saliva was again spun at 18,407 × g at 4°C for 20 min. Supernatant and pellets from saliva were tested for SVV by qPCR and no SVV was found in the supernatant. As such, for all study samples following centrifugation, all but 200 μl of the supernatant was decanted, the cell pellet was resuspended, and then placed on ice for DNA extraction.

**Buccal cells—**A Cytobrush Plus brush (Cooper Surgical, Trumball, CT) was inserted into both the left and right cheek pouches of anesthetized monkeys, rotated 10 times at each site, removed, and inserted into a sterile tube and kept at 4°C until DNA extraction.

### DNA Extraction and qPCR Assays

**Saliva—**DNA was extracted from saliva using the QIA-Amp DNA kit (Qiagen; Germantown, MD) (Mehta et al., 2017).

**Buccal cells—**Brushes containing buccal cells were placed directly in the DNA extraction buffer for lysis and DNA extraction, using a Gentra Puregene Buccal Cell Kit (Qiagen, Germantown, MD), following the manufacturer’s instructions.

**PBMCs—**DNA was extracted from PBMCs using a Qiagen DNA extraction kit (Qiagen), as described (White et al., 2002a,b; Mahalingam et al., 2007). DNA concentrations were determined using a NanoDrop ND-1000 Spectrophotometer (NanoDrop Technologies, Inc. Wilmington, DE).

**Quantitative real-time polymerase chain reaction (qPCR)—**SVV copy number was measured by real-time qPCR performed on the salivary and buccal DNA (3.5 μl per reaction) and PBMC DNA (100 ng per reaction) with TaqMan^®^ 7900, using fluorescence-based amplification (Applied Biosystems, Inc, Life Technologies, Grand Island, NY). Primers and Taqman probes specific for the SVV open reading frame (ORF) 61 were used for real-time qPCR as described (Cohrs et al., 2008) and have been our lab standard for all SVV diagnostics (Mahalingam et al., 2007, 2010; Traina-Dorge et al., 2015, 2019). Briefly, final concentrations of primers and probes in PCR were 500 and 250 nM, respectively. SVV ORF-61 sequences (forward primer 5′-ACACAGCGCTAATGAGAAGCC-3′; reverse primer 5′-GAAAGACGCTGCTGTTGTCG-3′; probe 5′-FAM/CAACCCCGCGTGTTGGCCC/3BHQ-3′). The presence of DNA in samples was confirmed by amplification of the cell gene, glyceraldehyde 6-phosphate dehydrogenase (GAPDH) using primers specific for GAPDH DNA (forward primer 5′-CAAGGTCATCCATGACAACTTTG-3′; reverse primer 5’GGCCATCCACAGTCTTCTGG-3′; probe 5′-ACCACAGTCCATGCCATCACTGCCA-3′). A standard curve was generated for GAPDH amplification using 0.1, 1.0, and 10 μg uninfected Vero cell DNA as substrate (Supplementary Figure 1). PCR conditions were as follows: 95°C for 20 s followed by 40 cycles of 95°C for 1 s and 60°C for 20 s.

Each animal sample was analyzed in a clean PCR room in triplicate and the copy numbers were averaged. Samples were only confirmed positive if two or three of the triplicate assays were positive. To generate standard curves for amplification with SVV ORF61 primers and probes, serial dilutions of wtSVV bacmid DNA (Gray et al., 2011) ranging from 1 to 10^8^ copies were prepared in a background of 20 μg/ml of herring sperm DNA and included a no DNA control. Any possible contamination showing previously amplified fragments are eliminated. Based on this SVV DNA standard curve, the sensitivity of our assay is 1–10 copies of SVV DNA. All PCR assays were carried out blinded with respect to identification of clinical specimens and positive controls.

PBMC SVV copy numbers were calculated as SVV DNA copies per 100 ng PBMC DNA. The saliva and buccal sample materials were more limited and contained lower quantities of extractable DNA than found in PBMC samples. GAPDH DNA amplification in these samples was used to quantify cellular DNA. Saliva and buccal cell samples yielded 0.4–3,693 ng/ul of cellular DNA based on GAPDH detection. Further, SVV qPCR was performed on these same samples to calculate SVV copies per ng cellular DNA. Results were multiplied by 100 and reported as SVV DNA copies per 100 ng of saliva or buccal cell DNA.

## Results

**Figure 1:**
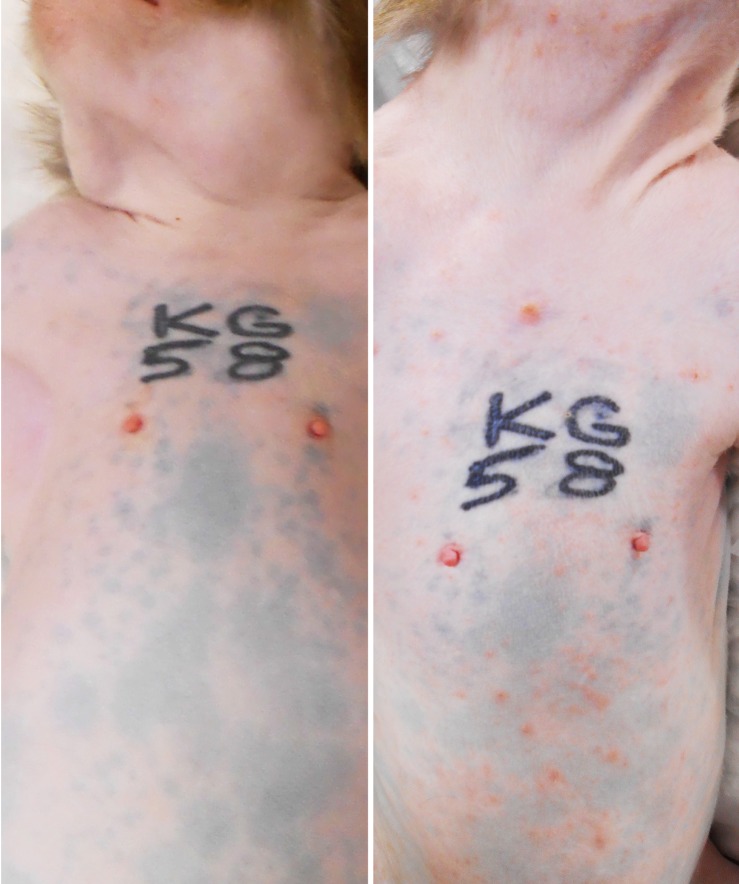
Primary varicella in experimentally inoculated rhesus macaque KG58. All monkeys developed typical varicella rash at 7–14 days post inoculation (dpi). Wide-spread generalized varicella rash of monkey KG58 on 9 dpi is shown on the neck, shoulders, torso, arms and abdomen (right panel) and compared with the pre-inoculation baseline skin from the same animal (left panel).

**Figure 2:**
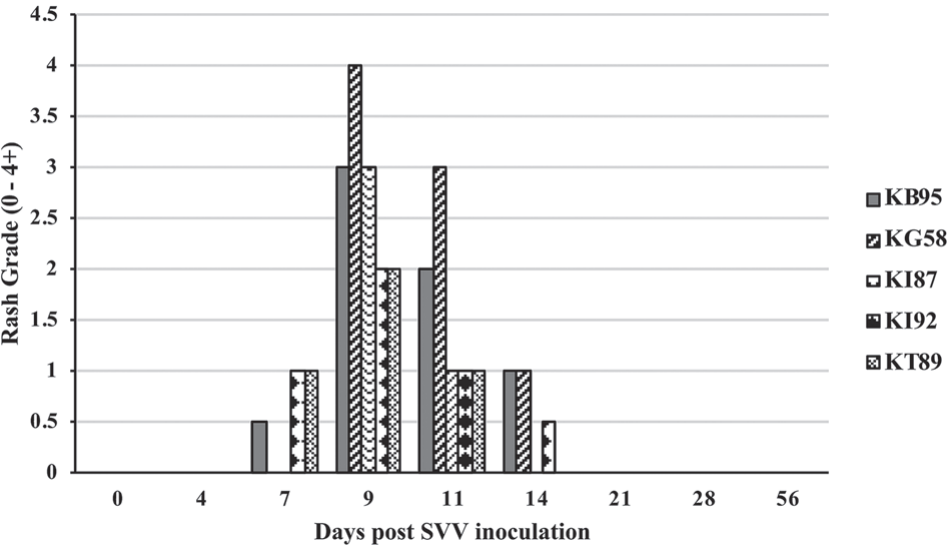
Rash after SVV inoculation. Monkeys were monitored for 56 days for varicella rash after SVV inoculation. All animals developed varicella with typical macular-papular-vesicular skin rash, graded (0 to 4+) during 0–14 dpi. Rash was first observed at 7 dpi, peaked at 9–11 dpi and resolved by 21 dpi.

All five monkeys inoculated with wtSVV developed typical acute varicella with viremia and macular/papular/vesicular skin rash in 1–2 weeks, Figure 1, shows skin in animal KG58 from d0 (left panel) and 9 dpi (right panel) showing typical acute varicella generalized rash. Figure 2 summarizes the extent of rash in all five animals. Monkeys KB95, KI92, and KT89 developed minimal 1+ skin lesions at 7 dpi, while all five monkeys developed moderate to severe, 2+ to 4+ peak rash at 9 dpi, with reduction to 1+ to 3+ at 11 dpi. Animals KB95, KI92, and KG58 still had minimal 1+ rash at 14 dpi. Varicella rash in all five animals was resolved by 21 dpi.

**Figure 3:**
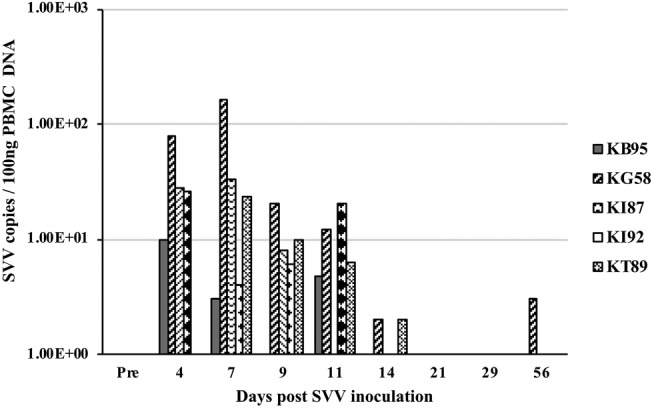
Viremia after SVV inoculation. Blood samples from the five intratracheally inoculated monkeys, were collected longitudinally at multiple times post inoculation. Peripheral blood mononuclear cells (PBMC) were isolated, DNA extracted, and SVV qPCR performed in triplicate on 100 ng DNA. Average SVV copy numbers/100 ng of PBMC DNA are shown.

At multiple timepoints after intratracheal SVV inoculation, PBMC were isolated and DNA was extracted and analyzed by real-time qPCR for the presence of SVV DNA. Four of five animals at 4 dpi and all five animals at 7 dpi, demonstrated peak viremia with 10–78 (mean = 35.5) and 4–161 (mean = 45.0) copies of SVV DNA per 100 ng of PBMC DNA, respectively. At days 9 and 11 dpi, only four of five animals had reduced levels of virus, with 0–20 (mean = 8.8 and 8.7, respectively) copies/100 ng PBMC DNA, while analysis at 14–56 dpi showed only sporadic 0–3 copies of SVV DNA/100 ng PBMC DNA (Figure 3).

**Figure 4:**
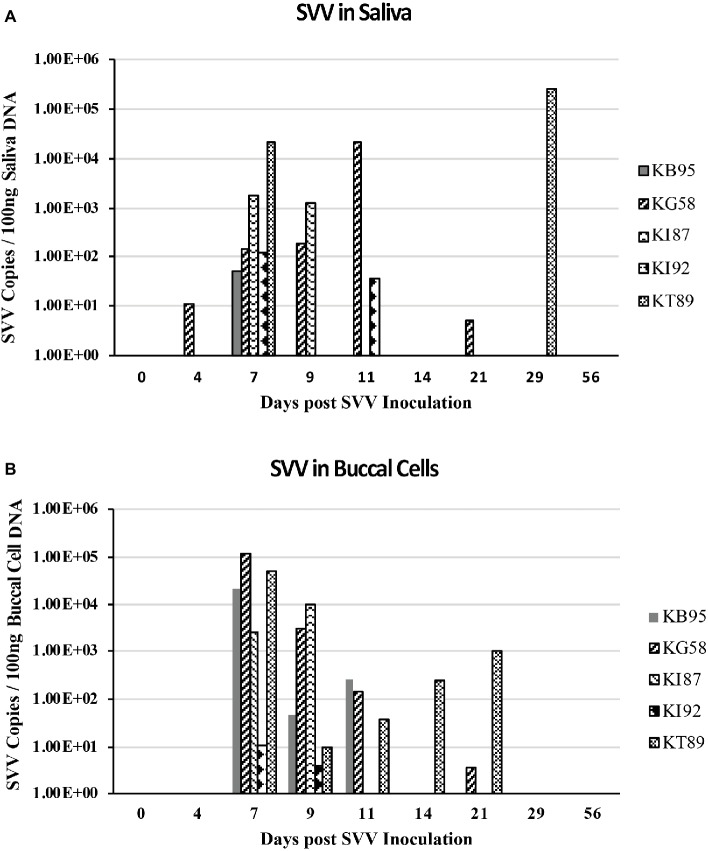
SVV DNA in saliva and buccal samples during acute infection. DNA was extracted from longitudinal saliva **(A)** and buccal **(B)** samples collected during acute SVV infection and analyzed by real-time qPCR for virus (SVV) and cellular (GAPDH) DNA. SVV qPCR was performed in triplicate on a standard 3.5 μl volume. Average SVV copy numbers/100 ng saliva DNA or buccal cell DNA for each sample are shown.

DNA from longitudinal samples of saliva and buccal cells were also analyzed by real-time qPCR for the presence of SVV DNA. At 7 dpi, SVV DNA was detected in all five animals, with 10^1^–10^4^ copies of SVV/ 100 ng of saliva DNA (mean = 4.6 × 10^3^, Figure 4A) and 10^1^–10^5^ copies SVV/100 ng of buccal cell DNA (mean = 3.8 × 10^4^, Figure 4B). At 9 dpi, saliva samples from two of the five monkeys contained 10^2^–10^3^ SVV copies/100 ng of saliva DNA (mean = 2.9 × 10^2^) while buccal samples from all five animals contained 10^1^–10^4^ SVV copies/100 ng of buccal cell DNA (mean = 2.5 × 10^3^). Similar to viremia, the SVV DNA present in saliva and buccal cells at 11–56 dpi was less frequent, with fewer animals positive for SVV DNA and in those monkeys, SVV copy numbers decreased from the earlier peak levels.

SVV DNA-positive samples as well as DNA copy number were higher in the majority of buccal samples than in saliva samples. On both 7 and 9 dpi, 9 of 10 buccal samples from monkeys: KB95, KG58, KI87, and KT89 had more copies of SVV DNA than did saliva samples (Figures 4A,B). Higher SVV DNA copy numbers in buccal samples than in saliva samples were also observed in monkey KT89 at: 4,11,14, and 21 dpi, showing SVV DNA-positive buccal cells with 1, 37, 250, and 1,030 copies of SVV DNA/100 ng buccal cell DNA, respectively, while their corresponding saliva samples were negative. Buccal samples from monkey KB95 obtained at 11 dpi had 256 copies SVV DNA/100 ng saliva DNA, while the matching saliva sample was negative. Saliva samples showing higher SVV copy numbers than in buccal samples were also seen, but less frequently. For example, the saliva sample from monkey KG58 obtained at 11 dpi contained 2.1 × 10^4^ copies of SVV DNA/100 ng saliva DNA compared to only 143 copies of SVV DNA/ 100 ng buccal cell DNA in the buccal sample obtained at that time. Monkey KI92 had SVV DNA-positive saliva at 7 and 11 dpi, with 119 and 37 copies of SVV DNA/100 ng saliva DNA, respectively, while the matching buccal samples had 11 and 0 copies of SVV DNA/100 ng buccal cell DNA, respectively. At 29 dpi, saliva from monkey KT89 also showed high levels (2.6 × 10^5^ copies of SVV DNA/100 ng saliva DNA), while the matching buccal sample was negative.

## Discussion

After SVV experimental inoculation of rhesus macaques, all monkeys became viremic (PBMC) and developed typical varicella rash within the first 2 weeks, as previously shown (Messaoudi et al., 2009; Ouwendijk et al., 2013). In our study, viremia reached peak levels at 4–7 dpi while rash presented later, with peak levels at 9-11 dpi. Despite viremia in four of five animals on 4 dpi, the saliva from only one of the five animals was SVV DNA positive while all buccal samples were negative, suggesting a possible non-hematogenous route for dissemination into the oral mucosa or delay in replication within epithelial cells. Our study demonstrated positive shedding of SVV into the oral cavity of non-human primates, as is also shown in human VZV acute infection of adults (Mehta et al., 2012), patients with zoster (Cohrs et al., 2008, Mehta et al., 2008, 2013, 2017, Depledge et al., 2011, Nagel et al., 2011, Gershon et al., 2015), and patients with zoster sine herpete (Furuta et al., 2001; Gershon et al., 2015).

We tested both saliva and buccal cells for SVV DNA to assess whether epithelial cells would provide a more reliable source of material for analysis from anesthetized animals. Epithelial cells are susceptible target cells of SVV infection and likely the source of virus in saliva. Volumes of saliva >1 ml are more difficult to reproducibly obtain from anesthetized animals compared with 2–3 ml saliva yields from human subjects (Mehta et al., 2013, 2017). Peak SVV DNA copies were present in both 7 and 9 dpi saliva and buccal samples. Although some discordances were observed, numbers of SVV DNA-positive samples and SVV copy numbers were generally greater for buccal cells than saliva samples, possibly due to the larger cellular yield of buccal samples than that obtained from saliva samples.

Our results clearly demonstrate SVV shedding in the oral cavity during acute SVV infection. Peak SVV copy numbers at 7 dpi, in both saliva and buccal samples parallels viremia, indicating that both sample types are relevant for diagnosis of SVV infection after experimental inoculation. Overall, the SVV DNA yield from buccal samples was higher than that obtained with saliva samples from anesthetized animals.

## Data Availability

All datasets generated for this study are included in the manuscript and/or the Supplementary Files.

## Ethics Statement

All animal housing and procedures were in accordance with the recommendations of the US Department of Agriculture Animal Welfare Act regulations, the *Guide for the Care and Use of Laboratory Animals,* and regulations of the Association for Assessment and Accreditation of Laboratory Animal Care (AAALAC). All experiments were reviewed and approved by the Tulane National Primate Research Center (TNPRC) Institutional Animal Care and Use Committee (IACUC), prior to the start of the study.

## Author Contributions

All authors contributed to the research as well as the writing of the manuscript.

### Conflict of Interest Statement

The authors declare that the research was conducted in the absence of any commercial or financial relationships that could be construed as a potential conflict of interest.

The handling editor declared a past co-authorship with one of the authors RM.

## Supplementary Material

SUPPLEMENTARY FIGURE 1 Efficiency of GAPDH DNA amplification in real-time qPCR. Primers and TaqMan probes specific for GAPDH were used for real-time qPCR using 0.1, 1.0 and 10 μg of uninfected Vero cell DNA as substrate, as detailed in Material and Methods. The threshold cycle (*C_T_*) values for each concentration from triplicate samples were plotted against the copy numbers. The slope of the curve and the efficiency of amplification (*R*^2^ value and percent efficiency) were calculated.
