# Supplementary figures and images for: Simian Varicella Virus DNA in Saliva and Buccal Cells After Experimental Acute Infection in Rhesus Macaques
Source: Front Microbiol. 2019 May 9;10:1009. doi: 10.3389/fmicb.2019.01009 (PMC6520666; doi:10.3389/fmicb.2019.01009)

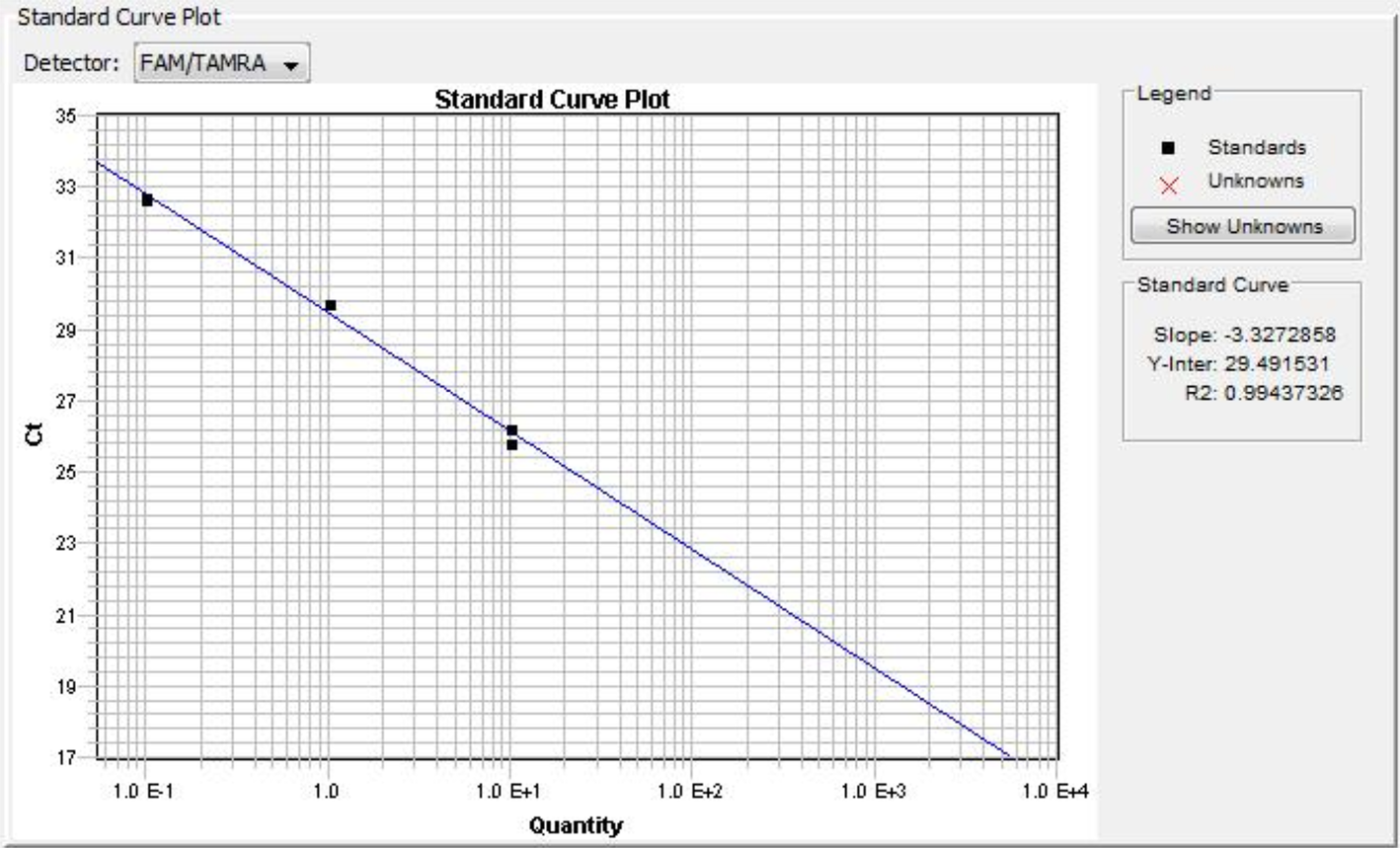

Supplement: SUPPLEMENTARY FIGURE 1 — Efficiency of GAPDH DNA amplification in real-time qPCR. Primers and TaqMan probes specific for GAPDH were used for real-time qPCR using 0.1, 1.0 and 10 μg of uninfected Vero cell DNA as substrate, as detailed in Material and Methods. The threshold cycle (CT) values for each concentration from triplicate samples were plotted against the copy numbers. The slope of the curve and the efficiency of amplification (R2 value and percent efficiency) were calculated. [file Image_1.tif]
